# Perspective on Micro-Supercapacitors

**DOI:** 10.3389/fchem.2021.807500

**Published:** 2022-01-11

**Authors:** Xiangfei Sun, Kunfeng Chen, Feng Liang, Chunyi Zhi, Dongfeng Xue

**Affiliations:** ^1^ Institute of Novel Semiconductors, State Key laboratory of Crystal Material, Jinan, China; ^2^ State Key Laboratory of Complex Non-ferrous Metal Resources Clean Application, Faculty of Metallurgical and Energy Engineering, Kunming University of Science and Technology, Kunming, China; ^3^ Department of Materials Science and Engineering, City University of Hong Kong, Kowloon, China; ^4^ Multiscale Crystal Materials Research Center, Shenzhen Institute of Advanced Technology, Chinese Academy of Sciences, Shenzhen, China

**Keywords:** micro-supercapacitors, integrated device, electrode materials, health monitoring, wearable electronic device

## Abstract

The rapid development of portable, wearable, and implantable electronic devices greatly stimulated the urgent demand for modern society for multifunctional and miniaturized electrochemical energy storage devices and their integrated microsystems. This article reviews material design and manufacturing technology in different micro-supercapacitors (MSCs) along with devices integrate to achieve the targets of their various applications in recent years. Finally, We also critically prospect the future development directions and challenges of MSCs.

## Introduction

The ongoing development of small electronic devices for telecommunication, microelectromechanical systems, and biomedical/environmental applications is creating a great demand for energy-autonomous systems (Shen et al., 2013; Li et al., 2016; Zhang et al., 2017a; Soam et al., 2017). Among many electrochemical energy accumulators such as lithium-ion batteries, fuel cells and supercapacitors (SCs), SCs have been widely studied by researchers due to their advantages such as fast charging and discharging speed, long cycle life and high power density (Wang et al., 2020a; Chen et al., 2020; Kumar et al., 2021; Wang et al., 2021). SCs, including electrochemical double-layer capacitors (EDLCs) and pseudocapacitors, show lower energy storage capability (ES ≤ 10 W h kg^−1^) compared to batteries (ES ∼ 180 W h kg^−1^). While SCs can be achieved a higher-power density (10 kW kg^−1^) (Guo et al., 2017; Chatterjee and Nandi, 2021). However, the shape of the device is greatly limited due to the unbending of the electrodes of traditional SCs. Moreover, the preparation of the electrodes involves metal collectors and binders, which also reduces the electrochemical performance of the SCs. Therefore, the development of a flexible and small supercapacitor matching with portable electronic products has become the development direction of the next generation of energy storage devices.

Micro-supercapacitors (MSCs) are the primary choice for advanced miniaturized energy storage devices due to their adequate power density and maintain a fast frequency response. In general, MSCs are sandwiched structures with sizes ranging from a few microns to centimetres. Thus, electrochemical properties available MSCs largely depend on the loading capacity and dispersion state of the active nano or micron particles. Compared with traditional rigid and bulky structures, their one-dimensional structures have various advantages (Zhai et al., 2020a):1) they have higher mechanical flexibility (which helps to withstand long-term and repetitive deformation); 2) Allow easy self-integration extension (which achieves the targets of their various applications); 3) Easy to fit into small spaces of different shapes (this brings versatility of the design); 4) It has the shape advantage of integrating with other one-dimensional devices (preferably for manufacturing multi-functional wearable systems).

**FIGURE 1:**
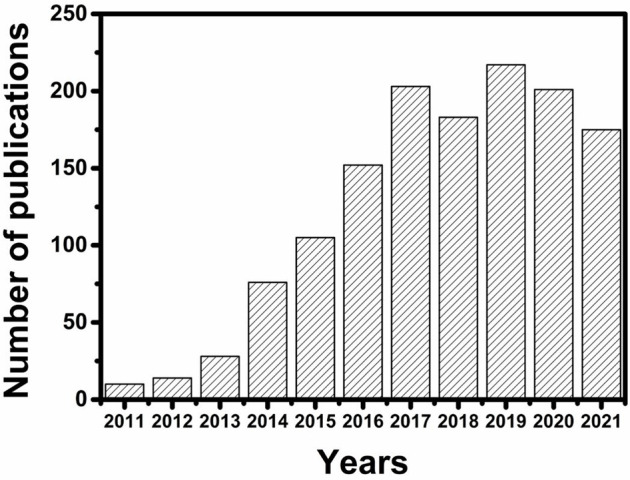
The number of article publication in the literature over the last decade. The values were obtained from a literature search using “micro-supercapacitors” (data source: web of science).

In the past few decades, the research on MSCs has developed rapidly and made great progress. Although some special aspects of MSCs can be found in the literature (as shown in Figure 1), there is still a great need for comprehensive reviews in time to introduce the latest developments in this exciting field. This article gives a comprehensive overview of the research progress of miniature SCs in the past severe years. First, the classification of SCs is briefly discussed. Next, the material design and manufacturing technology of MSCs in recent years are introduced. After these parts, it focuses on the latest progress in medical diagnosis with MSC. Finally, it summarizes the future development and existing problems of MSCs.

## Classification of Supercapacitors

SCs are defined as an electrochemical capacitor device have received greatest attention nowadays. According to previous literatures, the SCs have important features such as long life span (>10^5^ cycles) (Lu et al., 2017; Kumar et al., 2019; Zhai et al., 2020b) and higher energy density than electrochemical energy storage devices, and higher power density (5–15 kW kg^−1^) (Guo et al., 2017) as compared to battery and fuel cell. On the basis of charge storage mechanism, the SCs are be divided into two types including the pseudocapacitors and electric double layer capacitors (EDLCs) (Arbizzani et al., 1996; Hwang and Hyun, 2007; Miller et al., 2018; Seol et al., 2020).

**FIGURE 2:**
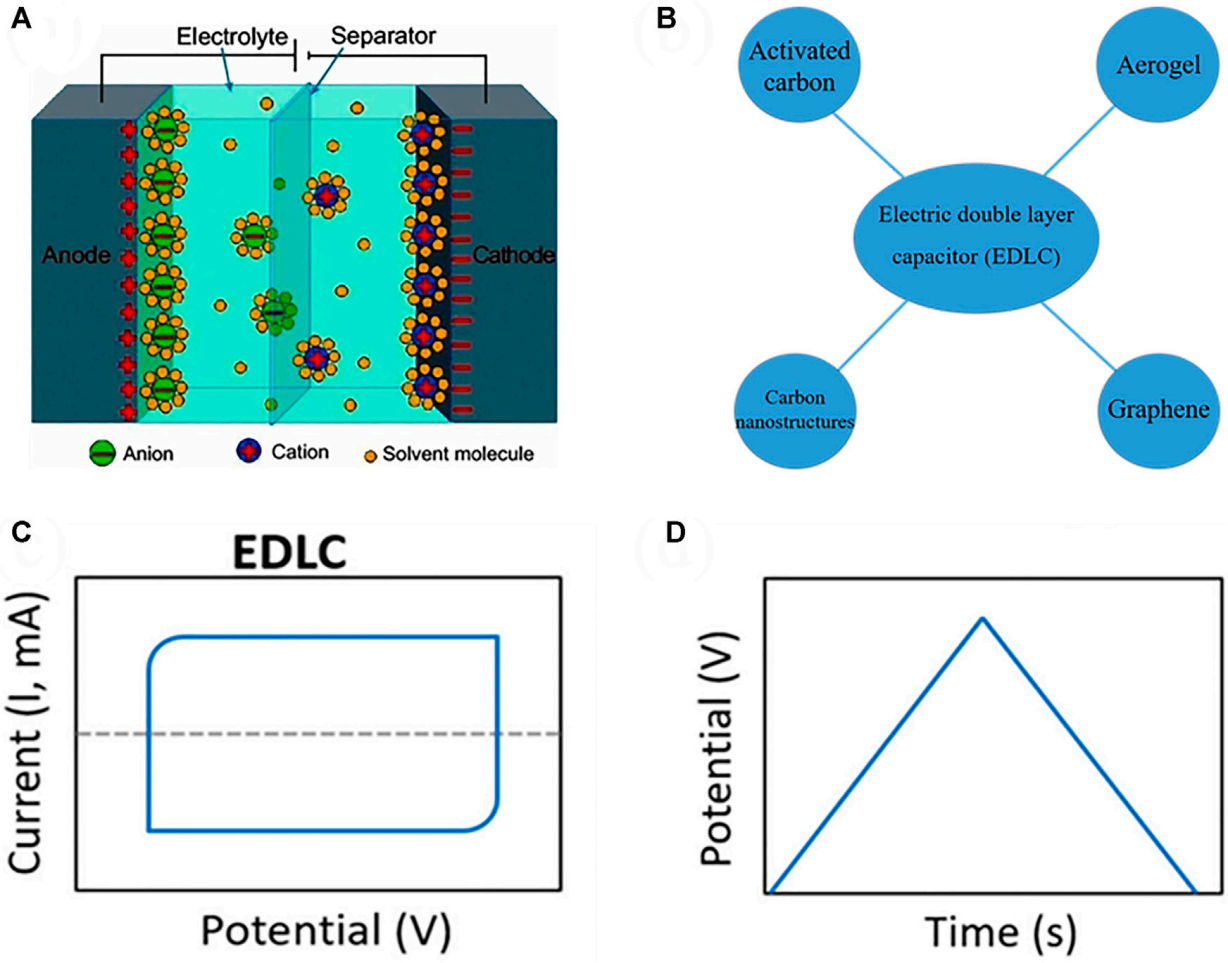
Schematic diagram **(A)** (Chen et al., 2017), material classification **(B)**, CV **(C)** and GCD **(D)** characteristic curves of EDLC (Jiang and Liu, 2019).

The EDLC uses the electric double layer interface formed between the electrode and the electrolyte to store charge (Morimoto et al., 1996; Zhang et al., 2009; Shao et al., 2020), and its structure is shown in Figure 2A. The separator separates the two electrodes. During charging, positive and negative charges accumulate on the surface of the two electrodes to form a capacitance. The accumulated charge during discharge returns to the electrolyte and generates a discharge current in the external circuit. At present, the electrode materials of a mainly include activated carbon (Wei et al., 2012; Wei and Yushin, 2012; Lei et al., 2013; Mori et al., 2019), carbon nanotubes (Zhou et al., 2014; Wang et al., 2016; Chang et al., 2018; Kshetri et al., 2018), carbon nanofiber (Liao et al., 2020), graphene (Kim et al., 2020; Wong et al., 2020), carbon materials (Xing et al., 2015; Xing et al., 2018) and aerogel (Fang and Binder, 2006; Zhang et al., 2006; Liu et al., 2018a), which have the characteristics of high conductivity, high strength, corrosion resistance, and high temperature resistance Figure 2B. The cyclic voltammogram (CV) curve (Figure 2C) and the galvanostatic charge/discharge (GCD) curve (Figure 2D) of the response of the EDLC exhibit rectangular and triangular shapes, respectively.

**FIGURE 3:**
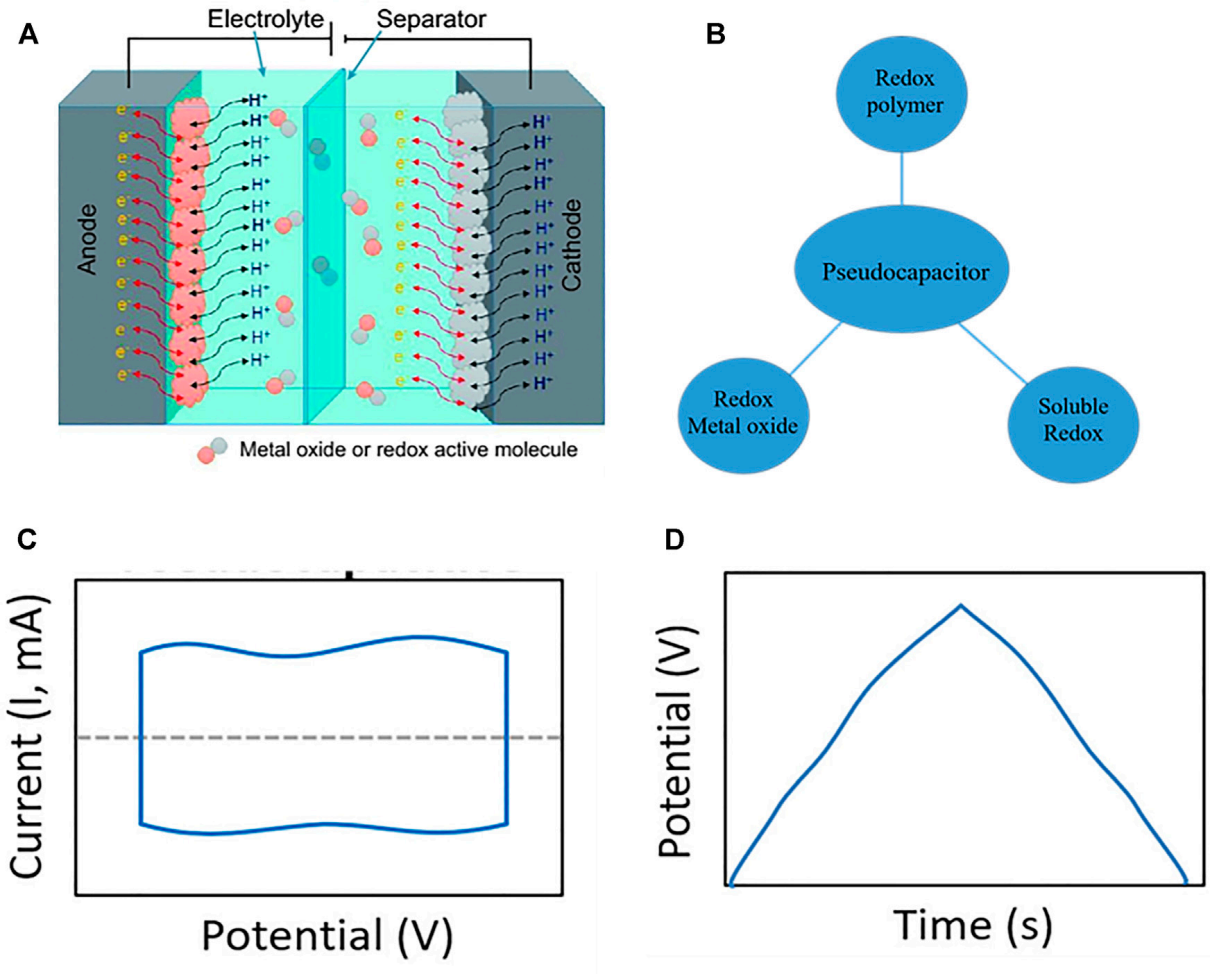
Schematic diagram **(A)** (Chen et al., 2017), material classification **(B)**, CV **(C)** and GCD **(D)** characteristic curves of pseudocapacitor (Jiang and Liu, 2019).

The capacitance of a pseudocapacitor comes from the oxidation-reduction reaction between the electrode material and the electrolyte. The electrode material is mainly metal oxide, metal-doped carbon and conductive polymer. Its structure is shown in Figure 3A (Chen et al., 2017). Electron transfer occurs during the capacitance generation process of pseudocapacitors. Although the electrochemical behavior is different from pure EDLCs, it is also different from batteries. Generally, redox polymer (Zhang et al., 2017b; Boota and Gogotsi, 2019; Witomska et al., 2019; Choudhary et al., 2021), redox metal oxide (Jiang et al., 2012; Chen et al., 2014a; Lee et al., 2016; Salunkhe et al., 2017; Wang et al., 2019) and soluble redox show pseudocapacitance (Figure 3B). The capacitance of a pseudocapacitor has a high degree of dynamic reversibility, and its CV curve (Figure 3C) presents a rectangular shape with redox peaks, which is a typical capacitive characteristic. The GCD curve (Figure 3D) has a charging and discharging platform.

Novel electrochemical energy storage technologies with enhanced energy capacity, and power capability are urgently needed. Supercapatteries can combine the merits of rechargeable batteries and SCs into one device (Archana et al., 2020; Yu and Chen, 2020). Recently, Xue et al. have developed colloidal supercapattery, which include colloidal electrode that can perform multiple-electron redox reactions and fast ion diffusion leaded to ultrahigh specific capacitance and fast charge rate (Chen et al., 2014b; Chen et al., 2015a; Chen et al., 2015b; Chen and Xue, 2017; Chen and Xue, 2018). Colloidal electrode materials include multiple varying ion forms, multi-interaction and abundant redox active sites. Colloidal electrode can skip over the material synthesis process to construct high-performance supercapattery. By only designing redox ions, the electrochemical performance of colloidal electrode is corresponding programmed. various redox cations with different oxidation states have shown promising application in colloidal supercapatteries, i. e. V^3+^, Mn^2+^, Fe^3+^, Co^2+^, Ni^2+^, Cu^2+^, Sn^4+^, Ce^3+^, Yb^3+^ and Er^3+^ ions (Chen et al., 2013; Chen et al., 2014c; Chen et al., 2014d; Chen et al., 2015c; Liang et al., 2018).

**FIGURE 4:**
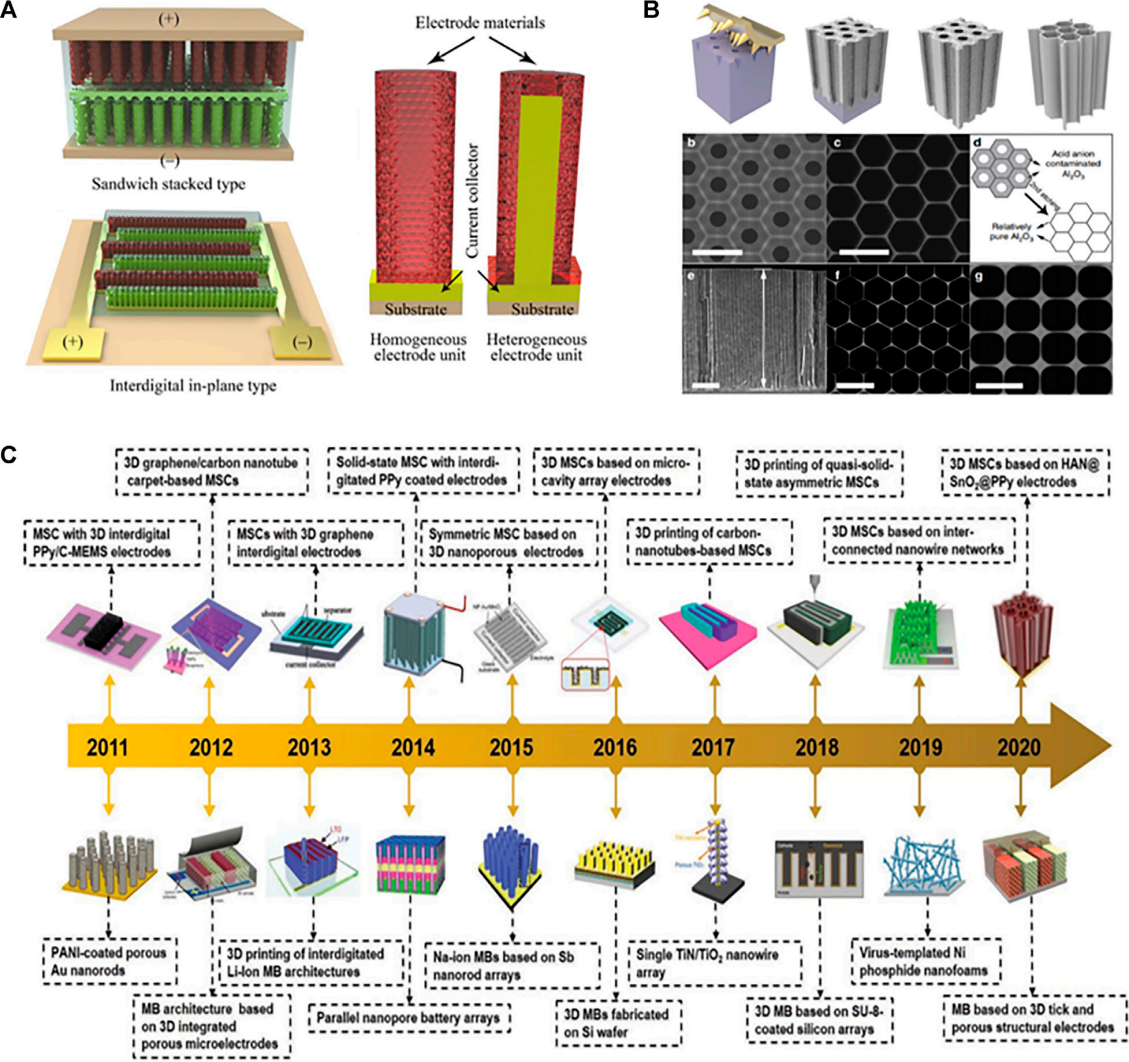
Schematic of 3D MSCs **(A)** (Liu et al., 2019a), Brief development featuring representative 3D architecture electrodes for MSCs **(B)** (Lei et al., 2020), Fabrication and structure of HAN **(C)** (Sha et al., 2021).

Due to its ultrahigh power density (>10 mW  cm^−2^), long lifespan (≥100 000 cycles), and remarkable mechanical flexibility, MSCs are recognized as the preferred miniaturized energy technology for a variety of autonomous electronic components (Zhao et al., 2017; Liu et al., 2019a; Bi et al., 2019). Figure 4A presents the 3D MSCs electrodes with sandwich and interdigital structure, respectively. Owing to the unique three-dimensional architectures (3D), 3D materials typically have a large specific surface area (>1,000 m^2^ g^−1^), which favors the accessibility of ion-active sites as well as improved ion and electron transport, hence facilitating reaction kinetics (Zhao et al., 2018a; Liu et al., 2019b; Lochmann et al., 2018). 3D MSCs have received a lot of attention in the last decade because of the obvious improved electrochemical performance of conventional SCs using 3D architecture electrodes, with the goal of developing micropower sources that meet both the dimensional and energetic requirements for on-chip integration (Figure 4C) (Sha et al., 2021). Particularly, significant progress has been made in the design and manufacturing of 3D architectural electrodes for the development of 3D MSCs (Liu et al., 2018b; Zhao et al., 2018b; Zhao and Lei, 2020). Lei et al. (Figure 4B) have designed the cell size of honeycomb monoliths (HMs) to the nanoscale, allowing for greater freedom in nanostructure design beyond their capacity for broad applications in many sectors (Lei et al., 2020). To avoid the formation of dense clusters of nanowires and nanotubes with high aspect ratios while meeting the criteria of high specific surface area and rapid ion transport dynamics, the cell size of conventional honeycomb monoliths should be decreased to the nanoscale level. Therefore, fabrication of microminiaturized cellular monomers-honeycomb alumina nanoscaffold (HAN) thus acting as a robust nanostructural platform for assembling the active material for MSCs.

## Materials for Micro-Supercapacitors

**FIGURE 5:**
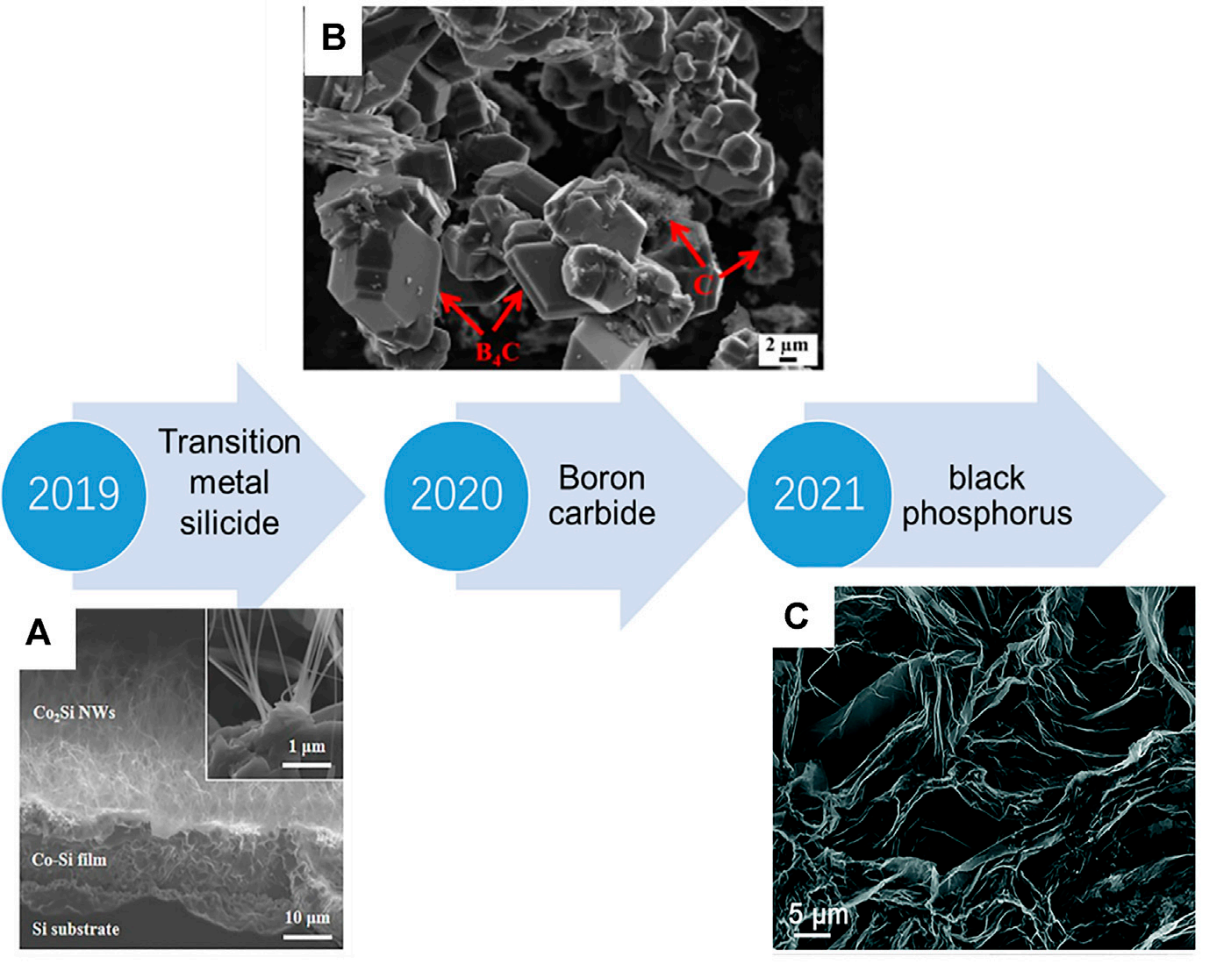
A brief timeline of the developments of promising of MSCs (In et al., 2008; Balcı et al., 2021; Yang et al., 2021a).

Materials for MSCs includes oxide, MXene, graphene, etc. Recently, novel materials are transition metal silicide, boron carbide (B_4_C), and black phosphorus (BP), Figure 5 shows the development timeline of micro-supercapacitor within 3 years.

**FIGURE 6:**
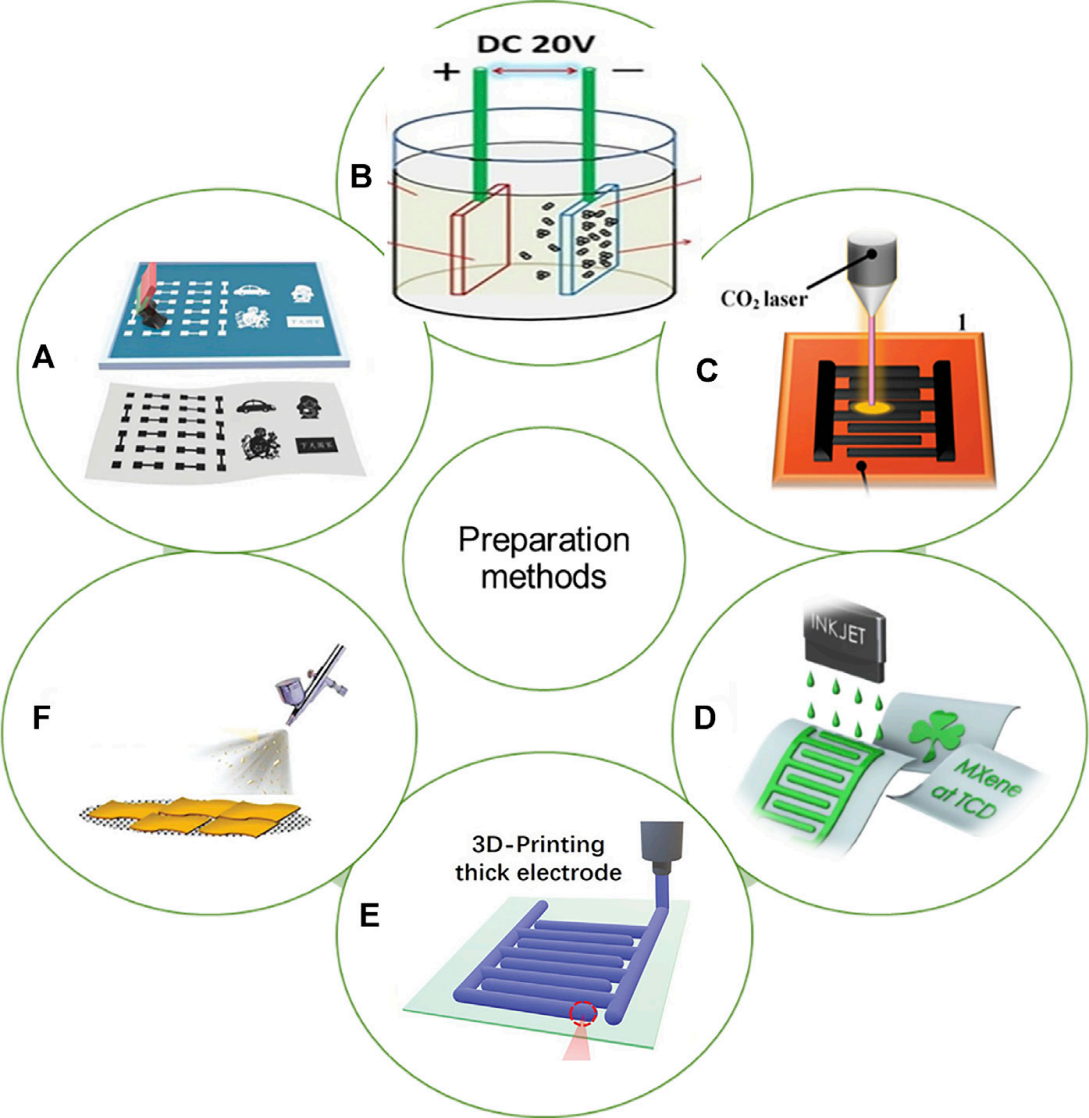
Schematic of the fabrication MSCs *via* different methods, **(A)** screen printing (Liang et al., 2020), **(B)** electrodeposition (Li et al., 2017a), **(C)** laser processing (Wang et al., 2020b), **(D)** ink printing (Zhang et al., 2019) **(E)** 3D printing (Li et al., 2020)and **(F)** spray-masking (Zhao et al., 2019).

### Transition Metal Silicide

Transition metal silicide is a type of intermetallic compound formed by non-metallic silicon atoms entering the crystal lattice of the transition metal. When Si atoms are inserted into the lattice of the transition metal, the d-electron bonding strength of the atoms becomes weaker, and the coupling between the energy state of Si and the metal orbital makes the electronic structure and geometric structure of the metal silicide diversified, thus having different due to the special physical and chemical properties of metals, such as high melting point, low resistivity, good heat transfer and excellent high temperature resistance, oxidation resistance, and corrosion resistance, it has been widely used in electric heating elements, integrated circuits and high temperature anti-oxidation coating and other fields. In addition, the transition metal silicides possess excellent interface properties with silicon and obtain low internal resistance because they are binder-free electrode materials (Ramly et al., 2018). It is worth noting that in addition to the above advantages, transition metal silicides also show intentional electrochemical properties, such as high theoretical energy capacity and low discharge potential. Up to now, Transition metal silicides are prepared by various methods such as arc melting, self-propagating high temperature synthesis, mechanical alloying (MA) (Okadome et al., 1995; Al-Joubori and Suryanarayana, 2016; Shin et al., 2017), Solid-state metathesis (Jacubinas and Kaner, 1993; Nartowski and Parkin, 2002), Molten salt reaction technology (Ma et al., 2004; Yang et al., 2004; Liu et al., 2013; Estruga et al., 2014), Microwave assisted synthesis (Vaidhyanathan and Rao, 1997; Zhang et al., 2014; Zhang et al., 2018), Chemical vapour deposition (CVD) (Seo et al., 2007; In et al., 2008; Higgins et al., 2010; Liu et al., 2012) and Solution synthesis route (Baudouin et al., 2012; Geaney et al., 2012; Galeandro-Diamant et al., 2019) (Figure 6). Each method described above has its own advantages and disadvantages. With the development of nanotechnology, methods such as CVD, solution synthesis routes, and polymer-derived pyrolysis have replaced traditional high-temperature methods. These methods can be used to produce high surface area materials. And control the size of the metal silicide particles to ensure the low cost, repeatability, high yield and scalability of the production technology.

### Boron Carbide (B_4_C)

Boron carbide (B_4_C), also known as black diamond, is the third hardest material known in nature with a hardness ranking only second to diamond and cubic boron nitride. Compared with other materials, B_4_C has very remarkable physical and chemical properties, such as extremely high hardness (27.4–37.7 GPa), lightweight (low density 2.5 g cm^−3^), high melting point (over 2,400°C), excellent corrosion resistance, high thermal stability, high elastic mode (460 GPa) and high neutron absorption cross section (Song et al., 2019). Recently, B_4_C nanowires, one kind of P-type semiconductor with a good ductility (No cracking within 70° bending angle) have become as one of the most promising candidates for electrode material. Compared with other 2D materials, few-layer BP always has a direct band gap, which can be adjusted between 0.30 and 2.2 eV by controlling the number of layers (Hao et al., 2016). B_4_C has been prepared by various methods, such as vacuum heating synthesis strategy (Chang et al., 2020), spark plasma sintering (SPS) (Xu et al., 2012; Moshtaghioun et al., 2013; Sahani et al., 2016). The two preparation processes are simple, short cycle, good repeatability, low cost, and conducive to large-scale production.

### Black Phosphorus (BP)

As an emerging two-dimensional material, black phosphorus (BP) have been proven to be an excellent electrode material for supercapacitor due to large spacing (5.3 Å) and weak van der Waals interactions between adjacent puckered layers (Dong et al., 2016), adjustable band structure (Zhu et al., 2020a) and high electrical conductivity (300 S m^−1^) (Yang et al., 2021a), good mechanical properties (166 GPa) (Jiang and Park, 2014). These characteristics enable BP to obtain ideal electrochemical performance. Compared with other two-dimensional materials, BP has adjustable band gap, high carrier mobility and optical and electronic anisotropy, which is widely used in transistors, photoelectronics and electrochemical energy storage devices (Kou et al., 2015; Ling et al., 2015; Liu et al., 2015). Atomically thick BP layers can be prepared via mechanical exfoliation (Chen et al., 2015d; Zhang et al., 2015), plasma etching (Jia et al., 2015), liquid-phase exfoliation (Yasaei et al., 2015; Bat-Erdene et al., 2017), electrochemical exfoliation (Erande et al., 2016), microwave-assisted, sheer exfoliation, CVD (Smith et al., 2016), mineralizer-assisted gas-phase transformation method (Xu et al., 2016) and wet chemistry method (Son et al., 2011; Du et al., 2012; Yoo et al., 2014). Mechanical exfoliation and plasma etching methods has many disadvantages, including poor repeatability, and low production yield. The electrochemical exfoliation method can only be carried out through experiments, and it has not been practically applied. Liquid-phase exfoliation, microwave-assisted, sheer exfoliation, CVD, mineralizer-assisted gas-phase transformation method and wet chemistry method can produce 2D nanomaterials with high efficiency, low cost, high yield, simple and large-scale production (Qiu et al., 2017).

The conventional MSCs are elongated stage adopted sandwich configuration structures with dimensions typically ranging from twenty to thirty of micrometers in thickness, and a length of several millimeters to meters. the stacked structure is prone to short-circuit phenomenon. The two electrodes must be kept at a proper distance to avoid short-circuiting of the device. At the same time, the electrode must load as much active material as possible to improve the energy storage capacity of the devices. At the same time, the electrode should be loaded with as many active materials as possible to improve the energy storage capacity of the device, both of which will increase the ion transport impedance and lead to a low power density; on the other hand, the sandwich laminated structure is too large to be integrated on microelectronic devices. Compared with the traditional sandwich structure supercapacitor, the planar micro supercapacitor is composed of cross-finger electrode. The gap between the two narrow forks is filled with electrolyte, which is conducive to the rapid transmission of electrolyte ions, thus achieving ultra-high power density. Thanks to the structure of the planar cross finger, the micro ultracapacitors not only maintain the ultra-long cycle stability, but also show better performance than the traditional sandwich ultracapacitors. Up to now, in-plane interdigital MSCs with excellent electrochemical performance are prepared by various methods such as photolithography (Kim et al., 2013; Wu et al., 2014; Diao et al., 2020), ink printing (Li et al., 2017b; Zhang et al., 2019), screen-pritting (Li et al., 2019a; Abdolhosseinzadeh et al., 2020; Liang et al., 2020), laser processing (Xie et al., 2016; Tao et al., 2019; Wang et al., 2020b), spray-masking (Wang et al., 2017; Xiong et al., 2019; Zhao et al., 2019), electrodeposition (Makino et al., 2013; Li et al., 2017a; Asbani et al., 2021) and 3D printing (Yu et al., 2017; Yu et al., 2018; Wang et al., 2020c). Each method of MSCs preparation has its own advantages and disadvantages, and most of the methods are difficult to satisfy the preparation of multiple MSCs simultaneously. Therefore, the selection of preparation method needs to consider many factors such as the type of electrode material. Finally, the thickness of the electrode materials prepared by these preparation methods is summarized in Table 1. It is satisfactory that the thickness of the materials prepared by all the preparation methods has reached the nanometer level.

**TABLE 1:** Thickness of electrode materials via different preparation methods.

Method	Materials	Min thickness	References
Photolithography	MXene	300 nm	Couly et al. (2018)
—	MXene/CNT	30 nm	Kim et al. (2021)
—	OAm@Ti_3_C_2_Tx	600 nm	Song et al. (2020)
Ink printing	Porous graphene microspheres	30 μm	Chang et al. (2021)
—	Graphene	30 nm	Sollami Delekta et al. (2019)
3D Printing	MXene	1.5 μm	Orangi et al. (2020)
—	MXene-AgNW-MnONW-C60	500 μm	Li et al. (2020)
—	Graphene−carbon black	35 μm	Zhang et al. (2021)
Spray-masking	MXene	20 nm	Li et al. (2019b)
Electrochemical deposition	Polyethylenedioxythiophene	600 nm	Kurra et al. (2015)
Laser processing	Carbon nanotubes (CNTs)@polypyrrole (PPy)	24.64 nm	Li et al. (2021)

## Application of Micro-Supercapacitors

MSCs have the potential of world market compared to other energy storage devices in various applications due to their superior performance with higher power density, fast rates of charge and discharge, and longer operating lifetime. MSCs are commonly applied in aerospace and automotive application, portable electronic devices, rollable displays, miniature biomedical equipment and other systems. We mainly introduce the application in medical diagnosis and industrial in recent years.

### Implanted Parts in the Body in Health Monitoring

In order to realize real-time health monitoring and accurate diagnosis and treatment, implantable medical electronic devices such as Pacemaker and Neurostimulator and other sensor systems have made rapid progress. In terms of the powering capability, implantable MSCs are generally in pursuit of high energy/power density, a fast charge–discharge rate and large mechanically deformable, thus biocompatible material design is a key component. Recently, Kim and co‐workers (Sim et al., 2018) reported a promising electrode material with a high flexibility and excellent electrochemical behavior. By trapping poly (3,4-ethylenedioxythiophene): poly (styrenesulfonate) and ferritin on multiwalled carbon nanotube, the fiber electrodes exhibited electrochemical stability and the electrical conductivity. Based on this, the capacitance loss of 16% in the mouse after 8 days, and fiber electrodes achieved excellent performance for validated biocompatibility for *In vivo* phenomena and cell response tests. In contrast to conventional battery/capacitor couple, a single material with the mixed electrochemical behavior for one device is of great improvement for the powering function within limited space.

**FIGURE 7:**
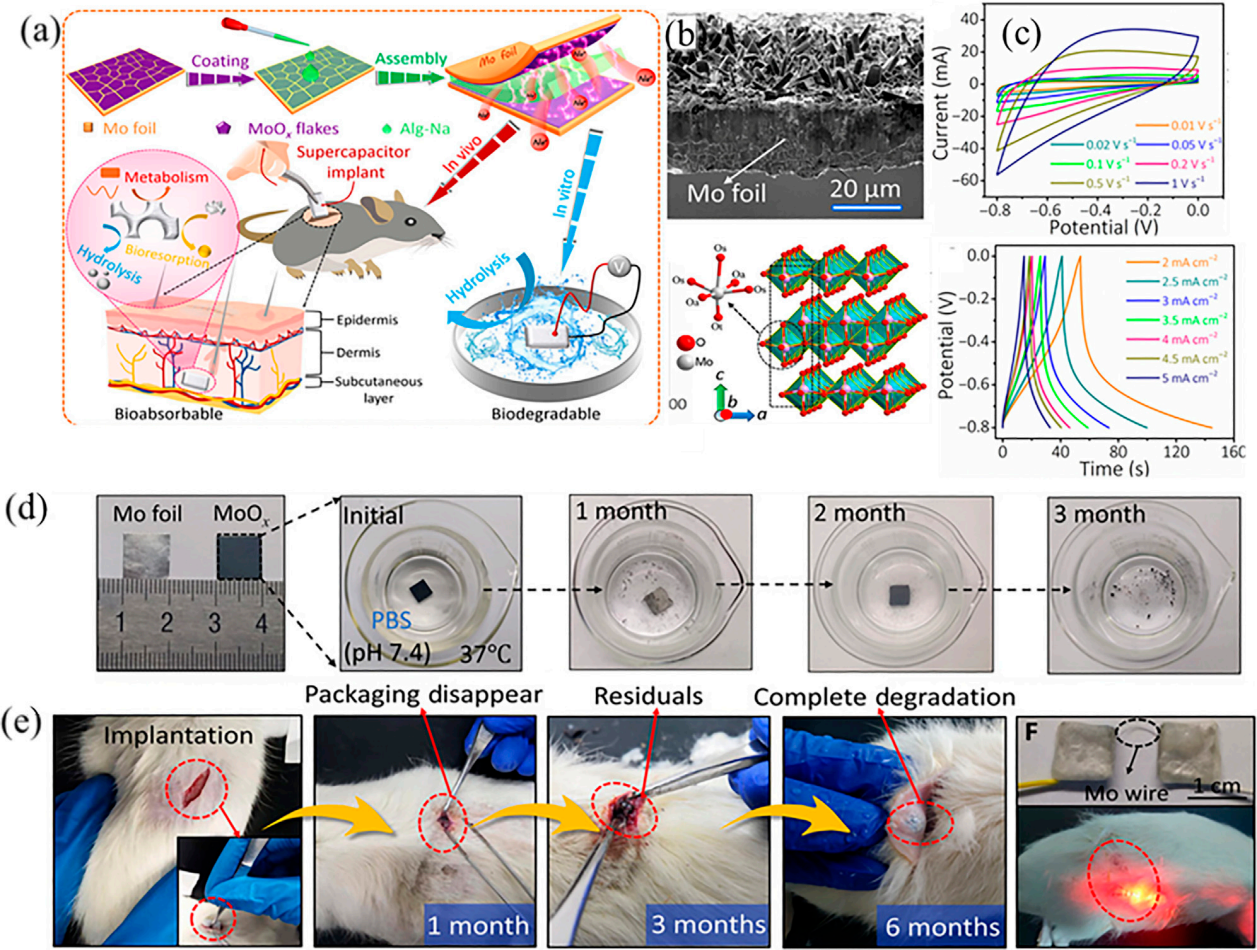
**(A)** Schematic representations of the synthesis procedure and applications of the biodegradable MSCs. **(B)** SEM image and crystal structure of MoOx electrode. **(C)** CV and GCD curves of MoOx electrode. **(D)** Digital pictures of the time-sequential dissolution of a single MoOx electrode (1 cm × 1 cm) immersed in PBS (pH 7.4) at 37°C. **(E)** *In vivo* degradation evaluation of the supercapacitor implant in the subcutaneous area of SD rats (Sheng et al., 2021).

Power biodegradable and implantable MSCs have attracted numerous investigations due to the needs for smart implantable medical electronics. Very recently, Sheng’s group (Sheng et al., 2021) reported on the fabrication of MoOx flake on water-soluble Mo foil by an electrochemical oxidation approach. The as-prepared MoOx-based supercapacitor delivers a high specific capacitance of 112.5 mF cm^−2^ at 1 mA cm^−2^ and a recoverable energy density of 15.64 Wh cm^−2^. The packaged device can work effectively for up to 1 month in a simulated body fluid environment (37°C, 0.1 mM PBS solution, pH = 7.4), and the length of the working life is controllable, which is comparable to the other reported materials and suggests that it is indeed a promising energy storage device in terms of energy storage properties. In addition to electrochemical performance, MoOx flake have shown the biodegradation behaviors and biocompatibility. As shown in Figure 7D, Sheng’s group further systematically studied its biodegradability by placing the MoOx electrode material in a neutral phosphate buffer filling solution. The study found that as time progressed, the MoOx flakes were first completely dissolved, and then the exposed Mo foil appeared to be cracked and corroded and decomposed into black powder. The above test is only a verification process, and the live implantation needs to be further proved its effectiveness. As shown in Figure 7E, 6 months after implanting the encapsulated biodegradable supercapacitor into a living mouse, the entire MSC device was completely dissolved, and the mouse did not show any inflammatory response. As we all know, molybdenum is an indispensable trace element to maintain the normal life activities of the human body, so the dissolution of electrode materials can also further provide the human body’s daily demand for Mo element.

Apart from that, practical applications in the human body is also important for the practical MSC. Gao and co-workers developed biocompatible and edible MSC using a laser scribing strategy, which has a highly flexible and excellent electrochemical performance in bioenvironment. (Gao et al., 2020) Composed of gelatin, edible gold, active carbon (AC) and agar aqueous electrolyte, the flexible edible MSC could be charged to 2 V even after soaking in simulated gastric juice for 28 min and degrade completely in a simulated gastric fluid for 40 min. Such flexible edible MSCs in a capsule was swallowed by testers, and the tester had no adverse reactions, which further proves the high safety of MSCs.

### External Fixation Components in Health Monitoring

Yun and co‐workers reported an integrated energy device for both generate photoelectric conversion, energy storage and sensing by fabricating MSC, strain sensor (SS) and commercial silicon-based solar cell (SC) on a single stretchable substrate. The MSC was obtained *via* photolithography and the e-beam evaporation. The integrated system would be charged when the MSC and SC were connected. The negative and positive charges generated from the DSSC part were transported toward the Ti wire and stored in the SC part shows the charging and discharging curves of the integrated optical charging system it was exposed to the light of the solar simulator. After being exposed to light, the voltage rapidly increased to 0.8 V within 2 s. During the discharge process, it reached the open circuit voltage (Voc) of the MSC after about 1.2 h. At the same time, in the mechanical stability test of the integrated system, after 1,000 cycles of repeated stretching (30% biaxial stretching), the initial capacitance only dropped by 2%, showing excellent electrochemical performance (Yun et al., 2018). Gong and co‐workers used solution treatment series solar cells combining perovskite solar cells (PSCs) and ternar organic solar cells (OSCs), and then integrate PSCs—OSCs series solar cells with solid-state asymmetric ultracapactors through solution treatment conducting polymer film to build a wireless portable solution treatment self-charging power pack. Upon exposed to light, the power conversion efficiency and energy storage efficiency were calculated to be 17.16 and 72.4%, respectively (Zhu et al., 2020b).

**FIGURE 8:**
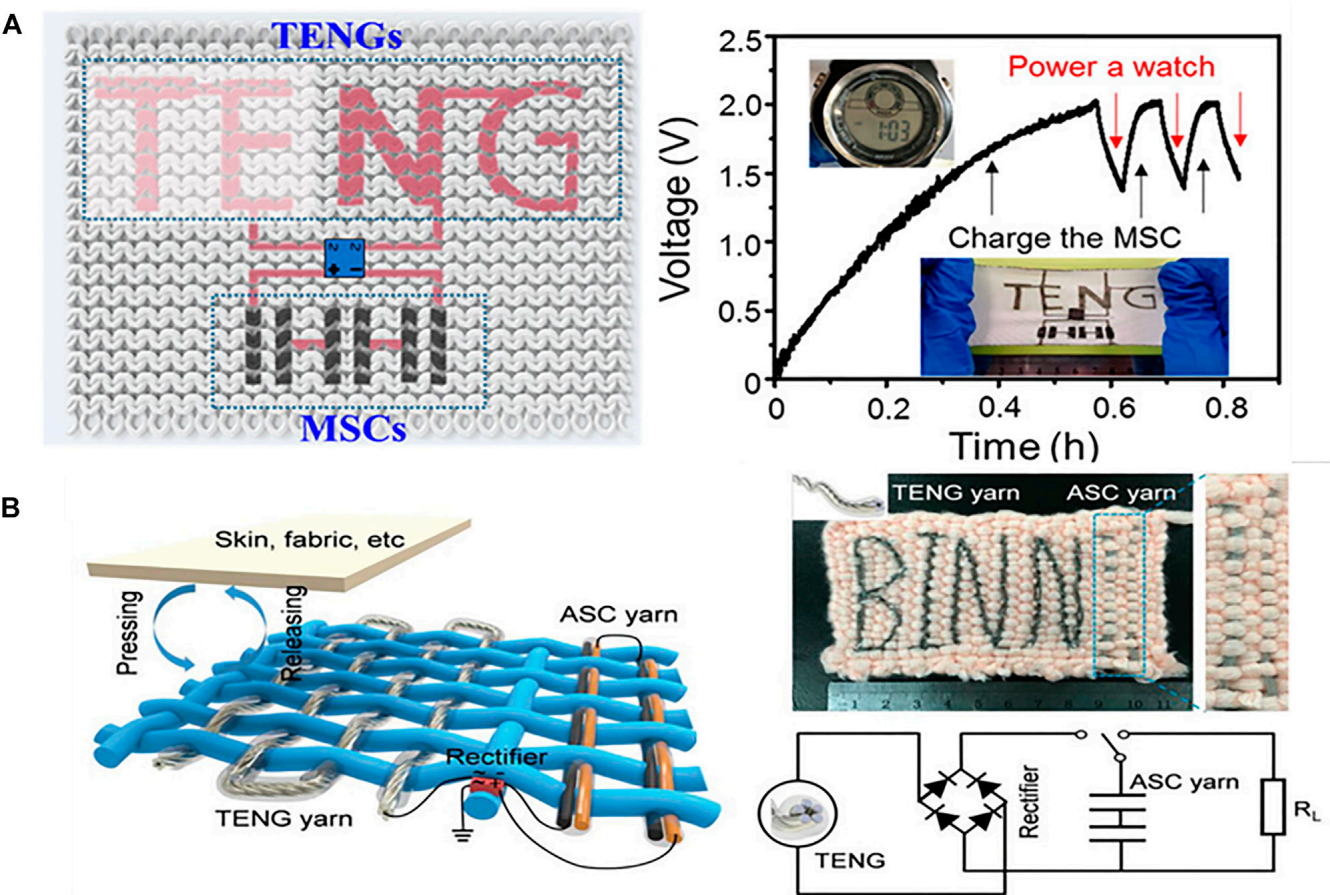
**(A)** Schematic illustration of the coplanar self-charging power textile (Cong et al., 2020). **(B)** Schematic illustration, photograph and Equivalent circuit of the self-charging power textile (Liu et al., 2019c).

These integrated devices can efficiently collect solar energy. However, the solar energy has the disadvantages of random, intermittent and dispersive, and the use of solar energy is largely affected by the weather, working conditions, etc. As a result, cong (Cong et al., 2020) and co‐workers recently developed a retractable coplanar self-charging system by integrating MSC prepared by chemical deposition and triboelectric nanogenerators into a common fabric, which can collect mechanical energy generated by human movement and converts it into electrical energy, resulting in a coplanar self-charging power textile (SCPT) as shown in Figure 8A. In this circuit, a bridge rectifier was used to charge the MSC with the current generated by the linear motor through beating of different frequencies. After 34 min of charging it to 2.0 V with a linear motor through a 4 Hz tapping, the self-chargeable power supply fabric can be used to power the electronic watch for 3 min. Liu and co-workers (Liu et al., 2019c) recently developed a yarn-based electronic system for efficient energy harvesting, conversion and storage (Figure 8B). To obtain the integrated energy textile, the negative electrode was first prepared by coating Cu-coated polyester yarn with rGO/CNT, and the positive electrode was prepared by growing Ni-Co bimetallic oxyhydroxide on the polyester yarn. Both the energy harvesting part and the storage part exhibited good performances, as confirmed from the experimental results. The triboelectric nanogenerators part exhibited a peak areal power density of 127 mW m^−2^. The MSCs adopted “sandwich” configuration and exhibited an areal energy density of 78.1 µWh cm^−2^. As a result, more complicated integrated system on an electronic textile is promising. Li and co-workers (Gao et al., 2021a) developed a Ti_3_C_2_Tx-derived iontronic pressure sensor (TIPS). The sensor consists of a floating electrode on the top and a microstructure PVA-KOH dielectric film. Ti_3_C_2_Tx has excellent electronic conductivity (∼10,000 S cm^−1^), electrochemical, optical and mechanical properties, and has better pseudocapacitance characteristics based on ion intercalation than traditional electric double layer electrode materials. Selected as the electrode of flexible PPS. The floating electrode design and the microstructured dielectric film further enhance the sensitivity. Benefiting from the synergistic effect between the electrode material and the device configuration, TIPS exhibits unprecedented ultra-high sensitivity (Smin> 200 kPa^−1^, Smax> 45,000 kPa^−1^), wide sensing range (20 Pa–1.4 MPa), and low the detection limit (LOD) is 20 Pa and has a stable long-term work durability of 10,000 cycles. These sensors can monitor physical activity and flexible robot tactile perception.

It is well known that body fluid is rich in chemicals such as different kinds of ions (Na, K, Zn, Fe), glucose and lactate. As non-invasive human body fluid monitoring provides health indicator information for human body, widespread research interest has been aroused by the related testing equipment. As far as sweat monitoring system is concerned, MCS for real-time health monitoring is generally in pursuit of high capacitance, biocompatible, flexible and long-term stability thus material design with highly compacted smarting sensing system being the key part (Li et al., 2019c; Lu et al., 2019). Recently, Lu and co-workers (Lu et al., 2019) developed a promising self-powered enzyme-free sensor arrays system with integrated flexible MSCs array. By coating the homogeneous mixed solution containing urchin-like NiCo_2_O_4_ and poly vinylidene fluoride on the lithographic PET, the symmetric MSCs exhibited a high capacitance of 0.067 F cm^−2^ at 1.2 mA cm^−2^ and a high energy density of 0.64 μWh cm^−3^ at 0.09 mW cm^−3^. Based on this, an excellent capacity retention of 96.6% was achieved for the MSCs after 20000 cycles. As-fabricated glucose sensor showed excellent response to glucose with concentrations ranging from 10 to 200 μM, and the detection limit of 10 μM was estimated. For the sweat sensors, the response concentration ranges of (Na) and (K) were 10–80 mM and 1–16 mM, respectively, indicating a high sensitivity to target solutions. As both the MSCs and the as-fabricated sensor showed good performances, a self-powered wearable monitoring system was designed to achieve sensitive and convenient body fluid monitoring, as exhibited in Figure 10B.

### Industrial Applications of Micro-Supercapacitors

**FIGURE 9:**
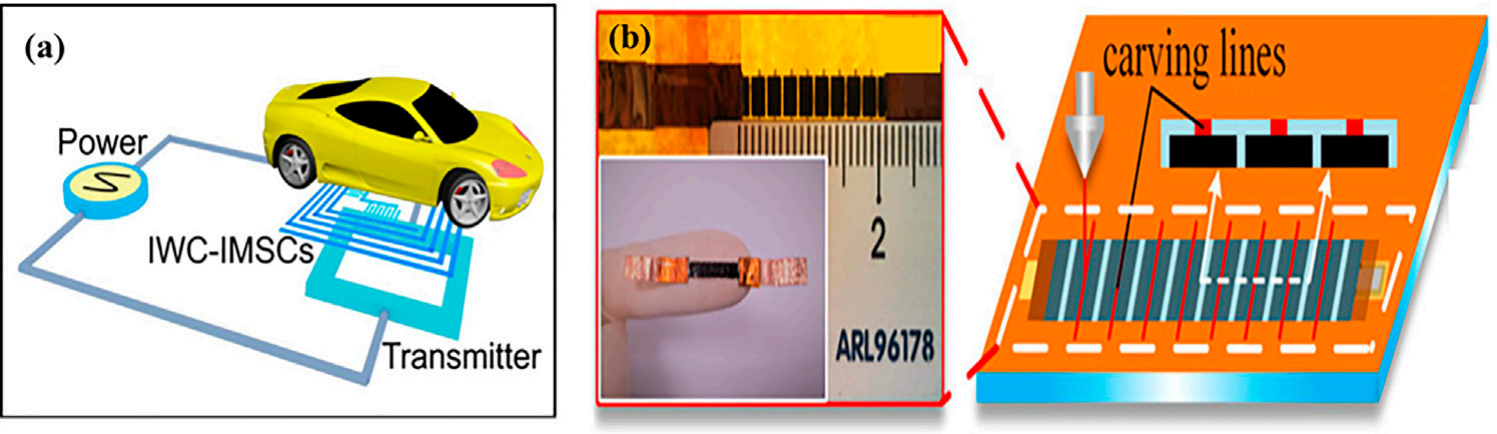
**(A)** Schematic illustration of operation of pure electric bus by the wireless charging energy storage microdevices (Gao et al., 2021b). **(B)** Schematic illustration and photograph of the flexible high voltage MSCs (Bai et al., 2020).

Various applications for MSCs have been reported in the previous literature and are brief reviewed in this here, including electric vehicle, micro-drones, micro-robots, railways and high-voltage power devices. Zhang et al. used the same material (graphite paper) to prepare the high-consistent material system with wireless coils and electrodes by laser etching approach. Due to the complete and unique circuit structure, the wireless charging system will operate at a high transferring power efficiency (52.8%), which affects the efficiency of the wireless charging energy storage microdevices. The electrochemical performances of a single MSC obtained the excellent capacitance of 454.1  mF  cm^−2^, higher than that of commercial Li-ion thin film batteries state-of-the-art conventional planar MSCs, superior than that of commercial thin film battery. And further when carefully integrated with the integrated wireless charging devices and MSCs, a microdevice integrating energy storage could drive the normal operation of pure electric bus, as demonstrated in Figure 9A (Gao et al., 2021b). Bai et al. (Bai et al., 2020) used laser radiation method to fabricate the flexible high voltage MSC (HVMSC) with series structure. As shown in Figure 9B, the device consists of the polyimide (PI) films, KOH electrolyte solution and graphene. Interestingly, the electrolyte layer on the surface of the laser-induced graphene is divided into several independent areas by the engraving line. In this process, conductive carbon black is formed, and the voltage of the MSCs is significantly increased through the alternate connection of electron and ion channels. Pang and co-workers (Pang et al., 2020)reported the application of MSCs for the forest fire monitoring and detection system as a power supply device. The author adopted a sliding friction electrification model, which consists of two elastically connected fixed and sliding sleeve layers. The breeze swayed the branches, and the tiny shaking caused by the branches can be effectively collected and converted into electrical energy by triboelectric nanogenerator, and stored in the integrated micro super capacitor to power the fire sensor. The relevant information and performance of MSCs produced in recent years are summarized in Table 2 for comparison.

**TABLE 2:** The relevant information and the performance metrics of the produced MSCs.

Active materials	Operating voltage	Specific capacitance (F g^−1^)	Energy density	Power density	Capacitance retention	References
MXene-AgNW-MnONW-C60	0–0.8 V	216.2 mF cm^−2^ (10 mV s^−1^)	19.2 μWh cm^−2^	58.3 mW cm^−2^	85% (10000 cycles)	Li et al. (2020)
GP-AC	0–3 V	63 mF cm^−2^ (0.5 mA cm^−2^)	58.4 μWh cm^−2^	0.57 mW cm^−2^	85% (10000 cycles)	Gao et al. (2021b)
Ni NPs	0–3 V	20.4 mF cm^−2^ (0.1 mA cm^−2^)	25.4 μWh cm^−2^	150 μW cm^−2^	89% (7,000 cycles)	Chae et al. (2021)
Laser irradiated graphene	0–1.2 V	2.32 mF cm^−2^ (10 μA cm^−2^)	0.46 mWh cm^−2^	0.57 W cm^−2^	∼100% (100000 cycles)	Kamboj et al. (2019)
Boron carbon Nitride	0–1 V	72 mF cm^−2^ (0.15 mA cm^−2^)	10 mW h cm^−2^ (0.15 mA cm^−2^)	487 mW cm^−2^ (1 mA cm^−2^)	∼100% (800000 cycles)	Karbhal et al. (2021)
MXene/SiC	0–1.6 V	97.8 mF cm^−2^ (1 mA cm^−2^)	8.69 μWh cm^−2^	0.8 mW cm^−2^	90% (10000 cycles)	Xia et al. (2022)
Ox-SWCNT/PVA/H_3_PO_4_	0–0.8 V	5–30 mF cm^−2^ (0.1 mA cm^−2^)	0.41 μWh cm ^−2^	0.37 mW cm^−2^		Yang et al. (2021b)
Mn/V oxide @MWCNT	0–2 V	11.8 mF cm^−2^ (0.2 mA cm^−2^)	6.58 μWh cm^−2^	200 μW cm^−2^	78% (5,000 cycles)	Park et al. (2021)
MXene/BC@PPy	0–1.9 V	388 mF cm^−2^ (1 mA cm^−2^)	145.4 μWh cm^−2^	0.36 mW cm^−2^	95.8% (25000 cycles)	Cheng et al. (2021)
Pristine graphene	0–1 V	1.57 F cm^−2^ (2 mA cm^−2^)	51.2 μWh cm^−2^	0.968 mW cm^−2^	87.6% (4,500 cycles)	Tagliaferri et al. (2021)

## Conclusion and Prospects

The research and preparation of MSCs are originated from the demand for miniaturized and integrated micro-energy storage system and evolve into multiple forms of applications. This review gives a comprehensive overview of the recent developments from electrode materials to application orientations. Integrated miniature SCs have made huge research progress in terms of size, flexibility, biocompatibility and degradability. Even though the application of integrated flexible MSCs in health monitoring and industrial applications has been successfully demonstrated, several challenges and problems have not been overcome.

1) The interference of monitoring environmental factors such as temperature, moderate and pH conditions on the health monitoring system needs to be resolved.
2) As an energy storage device, MSCs play an important role in ensuring the continuity and stability of work in the entire monitoring system. When designing devices, consider the use of miniature, flexible energy storage devices with larger capacitance, higher energy, wider working range, and longer service life.
3) The biocompatibility and degradability of implantable MSCs have been verified in small animal models, and the real human clinical trials are still generally verified.
4) There are differences in the performance of the MSCs, and the inconsistency of the voltages at the SCs will occur during the series use. If the MSCs is overvoltage or overcharged, its service life will be greatly reduced, and it will even cause permanent damage to the MSCs and greater voltage deviation.
